# MPE-YOLO: an efficient and lightweight model for maize plug-tray empty-cell detection and iOS deployment

**DOI:** 10.3389/fpls.2026.1886272

**Published:** 2026-07-22

**Authors:** Lei Xia, Liang Xu, Yujin Guo, Wenjie Kou, Yongjie Jia, Fuzhong Li, Xiaoying Zhang

**Affiliations:** Faculty of Software Technologies, Shanxi Agricultural University, Taigu, Jinzhong, China

**Keywords:** maize plug-tray, empty-cell detection, lightweight, deep learning, iOS-based application

## Abstract

**Introduction:**

Empty-cell recognition in maize plug trays is an important component of seedling quality monitoring in large-scale greenhouse nursery production, providing quantitative information for emergence assessment, plug-tray quality evaluation, and automated transplanting operations. Manual inspection is time-consuming and subjective, and automatic recognition remains challenging because greenhouse maize plug trays often contain neighboring-leaf occlusion and substrate-covered cell edges, while practical use also requires lightweight models suitable for mobile deployment.

**Methods:**

To address these challenges, this paper proposes MPE-YOLO, a lightweight YOLO26n-based detection model designed for greenhouse maize plug-tray environments. Depthwise separable convolution was incorporated into the backbone to reduce computational redundancy, while CGSB was introduced into the neck to reduce the number of model parameters and computational cost while maintaining feature representation. An EMA-enhanced C3k2 module, termed C3k2-EMA, was used to strengthen attention to empty-cell regions and suppress background interference. In addition, a dual-focusing loss function integrating Focal Loss and Wise-IoU v3 was designed to improve learning from hard-to-classify samples and targets with ambiguous boundaries. The optimized model was further deployed in an iPhone-based mobile application for portable empty-cell monitoring.

**Results:**

Experimental results from repeated tests showed that, on average, the proposed MPE-YOLO outperformed the original YOLO26n, improving precision and mAP@0.5 by 1.6 and 0.3 percentage points, respectively, while increasing inference speed by 16.0%. Stratified evaluation under occlusion and substrate-covering conditions showed that MPE-YOLO maintained better robustness in complex greenhouse plug-tray backgrounds. In empty-cell-free scenes, MPE-YOLO reduced the average number of false detections per tray from 3.1 to 1.3. The iPhone-based application also demonstrated the feasibility of using MPE-YOLO for portable detection.

**Discussion:**

This work provides an efficient and portable solution for maize plug-tray empty-cell recognition.

## 1 Introduction

Maize is one of the most extensively cultivated cereal crops worldwide and is integral to the animal feed supply, agri-food systems, and global food security (Széles et al., 2024). China is a leading maize-producing country, with production reaching 292 million tons in 2024, representing 23.98% of the global maize output (Liu et al., 2025b). In central Shanxi Province, a double-cropping system is being explored to increase annual grain productivity per unit area of arable land (Feng et al., 2023). In this context, greenhouse-based early seedling propagation can reduce the field occupation period after transplantation, thereby improving the temporal coordination between successive crops. Plug-tray propagation has been widely adopted in greenhouse seedling production because it enables uniform emergence and facilitates mechanical operations (Liu et al., 2023). Nevertheless, missed sowing, seedling mortality, and early growth abnormalities can generate empty cells within plug trays during production (Li et al., 2024b). Rapid and accurate recognition of empty cells can provide basic information for seedling quality monitoring in large-scale greenhouse nursery production, while also supporting the determination of seedling picking positions during automated transplanting operations (Liu et al., 2024).

Currently, empty-cell inspection of seedling trays largely depends on manual visual assessment. In large-scale greenhouse propagation systems, this approach is labor-intensive, time-consuming, and prone to variations caused by the inspector’s experience, working speed, and tray arrangement. With advances in computer vision, deep-learning-based object detection has been increasingly applied to agricultural vision tasks, including seedling identification, seedling counting, and seedling growth assessment. Existing detection models used in agricultural object recognition can generally be divided into two- and single-stage frameworks. Faster R-CNN (Ren et al., 2017), a representative two-stage detector, has been widely adopted for seedling-related detection tasks. For example, improved Faster R-CNN models have been applied to maize seedling detection (Quan et al., 2019), cotton seedling counting (Jiang et al., 2019), and abnormal seedling identification in hydroponic lettuce (Li et al., 2021b), achieving accurate detection performance under different agricultural scenarios. These studies indicate that two-stage detectors can provide accurate object localization in complex agricultural environments. However, their candidate-region generation and subsequent classification–regression processes usually introduce higher model complexity and computational cost, which constrains their applicability to lightweight real-time empty-cell detection in maize plug-tray production systems.

In contrast, single-stage detectors of the YOLO series perform object classification and localization through end-to-end regression, providing advantages in terms of detection speed, deployment flexibility, and real-time inference (Sapkota et al., 2025b; Sapkota and Karkee, 2025). These characteristics make YOLO-based models well-suited to agricultural vision tasks that require efficient field or greenhouse deployment. For example, Jiang et al. (2026) developed lightweight YOLOv8n-LP and YOLOv11n-LP models for maize seedling counting and leaf-age monitoring under open-field conditions. Zhang et al. (2024) proposed Seedling-YOLO for field-based quality inspection of transplanted broccoli seedlings, including empty planting-hole detection. Liu et al. (2025a) developed Grain-YOLO for infield rice grain detection and successfully deployed the model on Android mobile devices. These studies indicate that YOLO-based models can provide a favorable balance between detection accuracy and computational efficiency, showing strong potential for agricultural mobile deployment. More specifically, YOLO-series detectors have been increasingly applied to plug-tray quality monitoring tasks, including missed-seed detection, empty-cell identification, defective seedling recognition, and missing-seedling localization. Li et al. (2024b) proposed an improved YOLOv8s model for identifying empty cells and defective seedlings in eggplant plug trays. Yan et al. (2023) used YOLOv5x to detect tomato seeds in plug trays and determined missed-sowing cells by matching the detected seed positions with plug-cell boundary coordinates. Shi et al. (2026) developed Light-YOLO-Pepper for missing-seedling detection in pepper plug trays. Yu et al. (2026) proposed SD-MSL-YOLO for vegetable plug-seedling detection using RGB-D information. These studies demonstrate the feasibility of YOLO-based methods for plug-tray monitoring.

However, existing studies have mainly focused on vegetable crops, such as tomato, eggplant, and pepper, or on replanting scenarios for potted seedlings. Moreover, some studies have addressed missed sowing detection after seeding, rather than empty-cell identification after seedling emergence. In greenhouse maize plug trays at later seedling stages, leaves from neighboring seedlings may extend into empty-cell regions and cause occlusion, while substrate covering along cell edges can weaken the visibility of plug-cell contours and empty-cell boundaries, making empty-cell detection more challenging. Recent imaging studies in other complex visual environments, including underwater image enhancement (Wang et al., 2023, 2024, 2026), remote sensing visual interpretation (Liu et al., 2026), and compact HFSWR-related visual analysis (Sun et al., 2024; Li et al., 2025, 2026), also suggest that robust perception under complex conditions often benefits from task-specific feature enhancement and structured representation. This further supports the need for targeted model adaptation in greenhouse maize plug-tray empty-cell detection. Therefore, empty-cell detection in greenhouse maize plug-trays requires a task-adapted lightweight detector that can maintain strong discrimination under complex backgrounds, remain robust to neighboring-leaf occlusion and substrate-covered cell edges, and support real-time inference and mobile deployment. YOLO26n (Sapkota et al., 2025a), the latest member of the YOLO series, provides a favorable balance between model size and detection performance and was therefore selected as the baseline. To meet these requirements, this study proposes MPE-YOLO, a lightweight detector for greenhouse maize empty-cell plug-tray detection. Through targeted feature enhancement, lightweight architectural design, and localization-loss optimization, MPE-YOLO improves empty-cell detection accuracy and inference efficiency in complex seedling-production environments. Furthermore, MPE-YOLO was deployed on an iPhone, and a mobile empty-cell monitoring application was developed to enable rapid, non-destructive, and portable detection of empty cells in greenhouse maize plug trays, thereby providing technical support for emergence assessment and plug-tray quality evaluation.

## 2 Materials and methods

**Figure 1:**
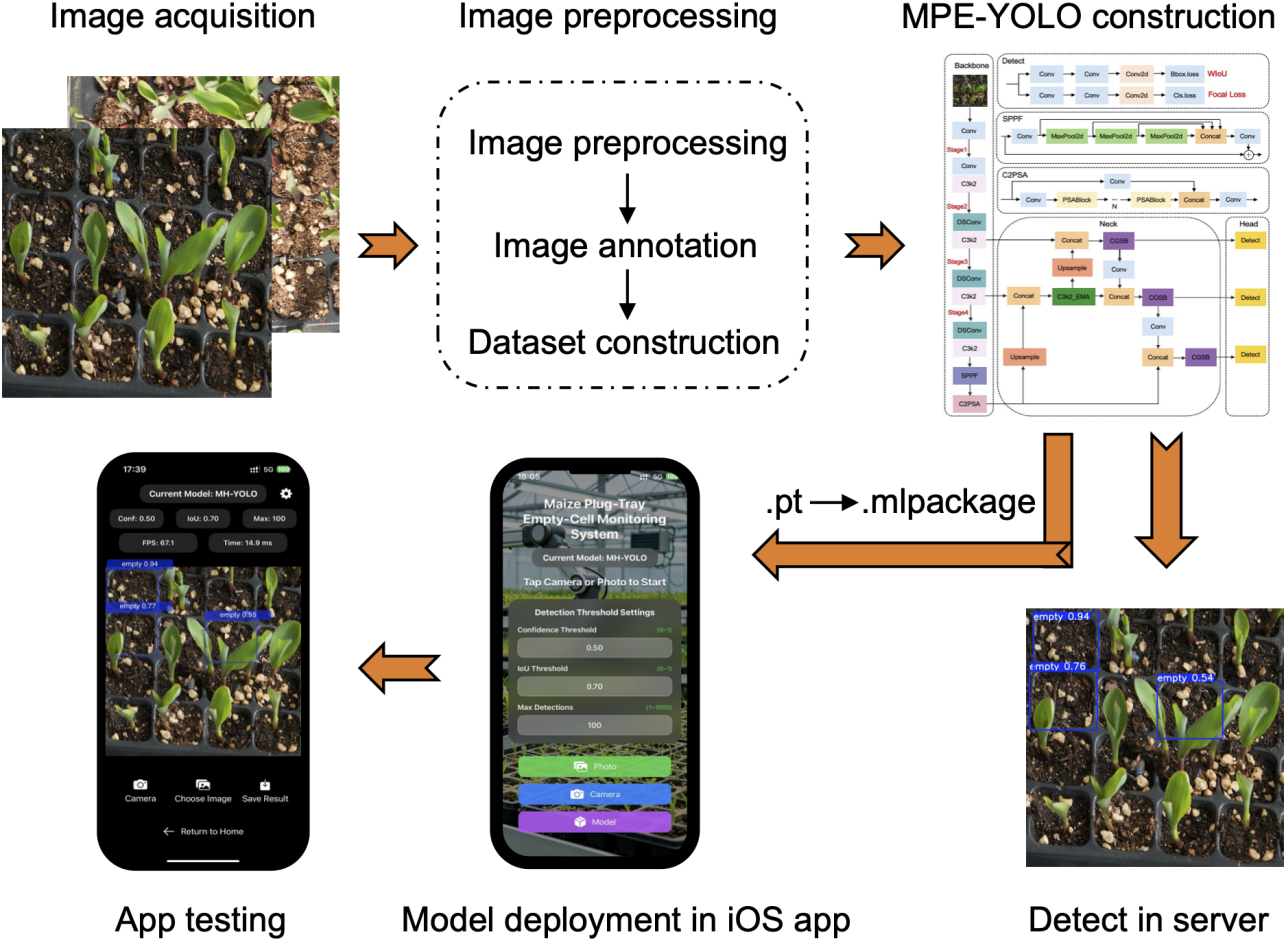
Overall framework for maize plug-tray empty-cell detection.

The overall framework of this study is summarized in **Figure 1**. The workflow consisted of maize plug-tray image acquisition, dataset annotation and preprocessing, MPE-YOLO model development, and mobile-device deployment.

### 2.1 Data acquisition and preprocessing

**Figure 2:**
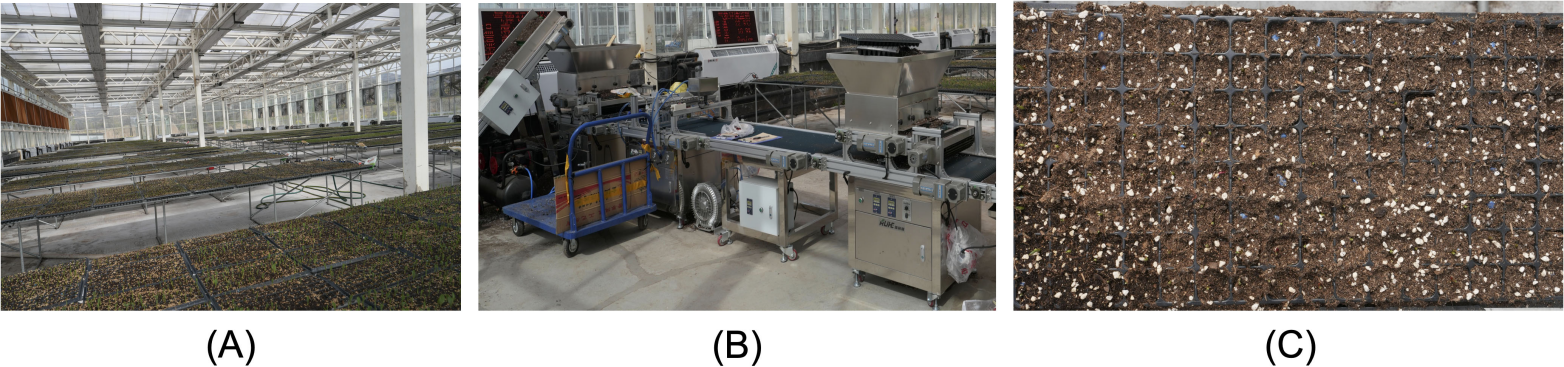
Data collection environment. **(A)** Experimental greenhouse. **(B)** Sowing machine. **(C)** Maize plug tray.

This study focused on empty-cell detection in greenhouse maize plug-tray seedling production, using Xianyu 335 and Linong 368 as the experimental maize cultivars. Data were collected from the Northern Fruit Tree and Seedling Breeding Base of Shennong Technology Group, Taigu District, Jinzhong, Shanxi Province, China (112.53°E, 37.34°N). The greenhouse environment used for maize cultivation is shown in **Figure 2A**. Images were acquired using a SONY A7R V digital camera with a resolution of 9504 × 6336 pixels. The maize seeds were automatically sown on March 25, 2025, using the seeding machine (**Figure 2B**) and planted in 16 × 8 plug trays (**Figure 2C**). Images were acquired on April 4, 7, and 10, 2025. During data collection, images were captured manually using a camera positioned approximately 50 cm above the plug trays.

To capture the heterogeneity of practical greenhouse production conditions, images were acquired under multiple environmental and phenological conditions, including different illumination periods, seedling developmental stages, and substrate moisture states. Image acquisition was performed during three time windows, namely 9:00-11:00, 13:00-15:00, and 16:00-18:00, to incorporate natural variations in solar illumination. The sampled seedlings covered the VE and V1 stages, where VE indicates seedling emergence above the substrate surface, and V1 denotes the one-leaf stage (Nleya et al., 2016). At the VE stage, seedlings are small and exert limited occlusion on neighboring plug cells, whereas at the V1 stage, increased seedling height and leaf expansion may cause leaves to extend into adjacent empty cells, resulting in partial occlusion. In addition, images were collected under relatively wet and moderate substrate conditions to account for moisture heterogeneity during greenhouse seedling production. Furthermore, because the trays were mechanically sown and simultaneously covered with the substrate, the boundaries between adjacent cells were partially obscured, producing discontinuous plug-cell contours and reducing boundary distinguishability. Together, these acquisition conditions increased the scene diversity and enabled the dataset to reflect the principal visual challenges of maize plug-tray empty-cell detection in practical greenhouse environments.

**Figure 3:**
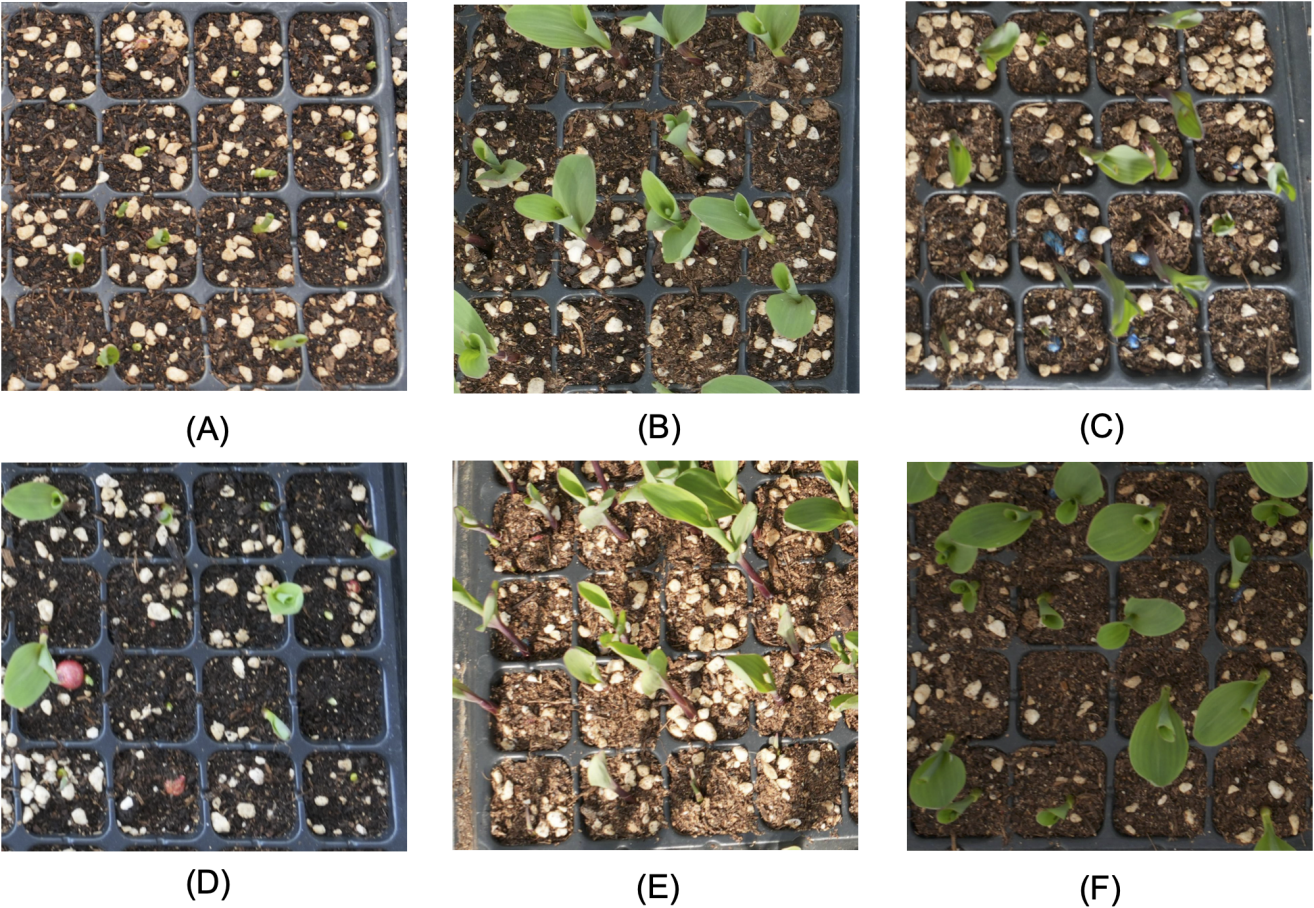
Representative examples of data diversity under different image acquisition conditions, including growth stages, substrate moisture levels, and lighting conditions. **(A)** VE stage. **(B)** V1 stage. **(C)** Moderate substrate moisture. **(D)** High substrate moisture. **(E)** Front lighting. **(F)** Backlighting.

**Figure 4:**
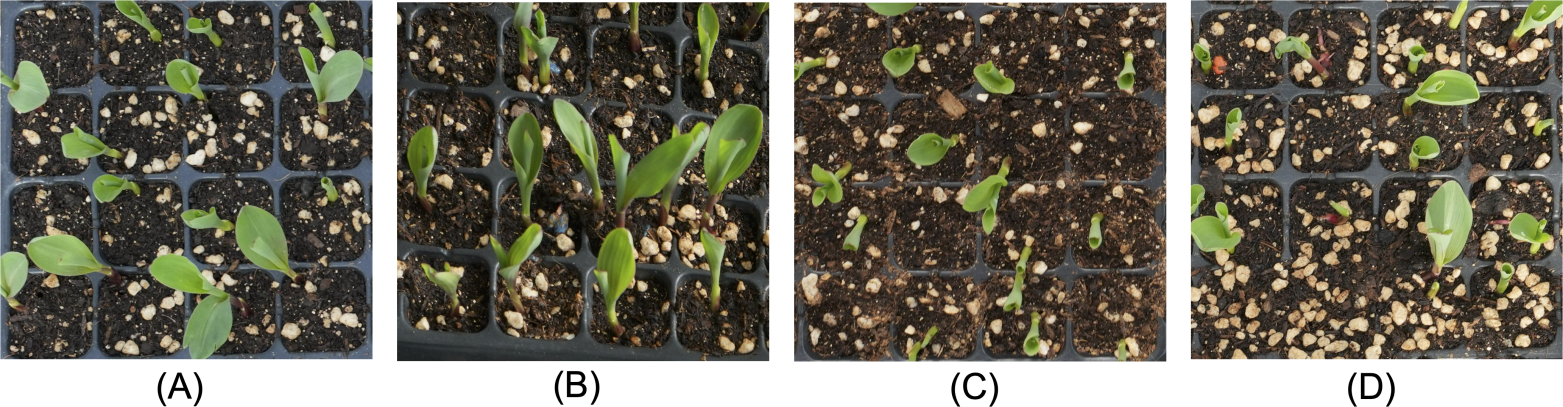
Representative examples of different complex background scenes. **(A)** Slight occlusion. **(B)** Severe occlusion. **(C)** Slight substrate covering. **(D)** Severe substrate covering.

Owing to the high resolution of the original images, a unified local cropping strategy was applied using a plug-tray grid unit as the basic cropping unit. During cropping, each subimage retained a complete 4 × 4 array of plug cells and contained at least one empty cell target. This procedure preserved the local structural relationships between empty cells and their surrounding complex backgrounds while substantially improving the efficiency of model training. Representative cropped samples covering different growth stages, substrate moisture states, and illumination conditions are shown in **Figure 3**. Although the dataset was collected across different maize seedling growth stages, acquisition times, and substrate moisture states, these factors mainly describe the data-acquisition conditions and may influence image appearance in a broader manner. In contrast, neighboring-leaf occlusion and substrate-covered cell edges can become two major sources of false detections and missed detections in practical greenhouse maize plug-tray seedling production because they directly affect the visibility, boundary clarity, and localization difficulty of empty-cell targets. Therefore, the stratified evaluation in this study was organized according to these task-relevant visual interferences rather than all acquisition-related conditions. Representative examples of these complex backgrounds are presented in **Figure 4**, including cases of slight occlusion, severe occlusion, slight substrate coverage, and severe substrate coverage.

### 2.2 Data annotation and dataset construction

To construct the empty-cell detection dataset for model training, all cropped image patches were manually annotated using LabelImg, and the annotations were exported in the YOLO format. Given that this study focused on the automated inspection of maize plug-tray seedling production, the annotation unit was defined at the plug-cell level. Specifically, plug cells containing either visible seeds or emerged seedlings were regarded as non-empty, whereas only plug cells without visible seeds or seedlings were labeled as *Empty*. Bounding boxes were delineated according to the complete region of each target plug cell to ensure consistency in annotation extent. For samples with ambiguous cell boundaries or weak seedlings, a unified decision rule was applied during annotation review to maintain labeling consistency. The dataset was then randomly divided into training, validation, and test sets at a ratio of 7:2:1. The number of samples in each subset is listed in **Table 1**.

**Table 1:** Number of maize plug-tray images and empty-cell counts.

Dataset	Number of images	Label counts for empty-cell
Training set	1321	3826
Validation set	377	1048
Test set	189	586
Total number	1887	5460

Given the regular grid layout and stable local spatial configuration of maize plug-tray images, data augmentation should increase sample diversity without substantially disrupting plug-cell boundary features or positional relationships among neighboring cells. Accordingly, this study avoided large-magnitude random geometric transformations and constrained the augmentation parameters within operations supported by the training framework, thereby establishing a plug-tray structure-aware augmentation strategy. Specifically, moderate photometric perturbations and restricted geometric transformations were introduced during training. Photometric perturbations were used to simulate illumination variations and local shadow interference under greenhouse conditions, whereas geometric transformations were used to account for slight scale changes and minor viewpoint deviations during image acquisition. This strategy improves model generalization while preserving the continuity of local plug-tray structures and the spatial discriminability of empty-cell targets. The specific augmentation parameter settings are listed in **Table 2**.

**Table 2:** Parameter settings of data augmentation strategies for plug-tray structural features.

Augmentation category	Description	Corresponding YOLO parameter	Parameter value
Color Distortions	Hue variation range	hsv_h	0.015
	Saturation variation range	hsv_s	0.4
	Brightness variation range	hsv_v	0.35
Geometrically constrained transformation	Scale variation range	scale	0.1
	Translation range	translate	0.05
	Rotation angle range	degrees	5
	Horizontal flip probability	fliplr	0.5
	Vertical flip probability	flipud	0

### 2.3 Architecture and deployment of MPE-YOLO

#### 2.3.1 Baseline YOLO26n

YOLO is a representative family of real-time object detection algorithms that converts object localization and classification into an end-to-end regression task through a single forward pass (Redmon et al., 2016). YOLO26, developed by Ultralytics, is the latest generation of the YOLO series and is designed for efficient real-time detection on edge and low-power devices. It introduces a streamlined detection pipeline with end-to-end NMS-free inference, thereby simplifying model export and improving its compatibility with deployment-oriented hardware platforms (Sapkota et al., 2025a).

YOLO26n is a nanoscale version of YOLO26 that provides a favorable balance between model compactness and detection performance. Its lightweight configuration makes it suitable for real-time inference on mobile devices, which is consistent with the deployment requirements for maize plug-tray empty-cell detection. Nevertheless, greenhouse plug-tray images contain neighboring-leaf occlusions and substrate coverage along plug-cell boundaries, which can reduce the discriminative capacity of the baseline model. Accordingly, YOLO26n was adopted as the baseline network in this study and targeted improvements were introduced to construct MPE-YOLO for accurate and efficient empty-cell detection under complex greenhouse conditions.

**Figure 5:**
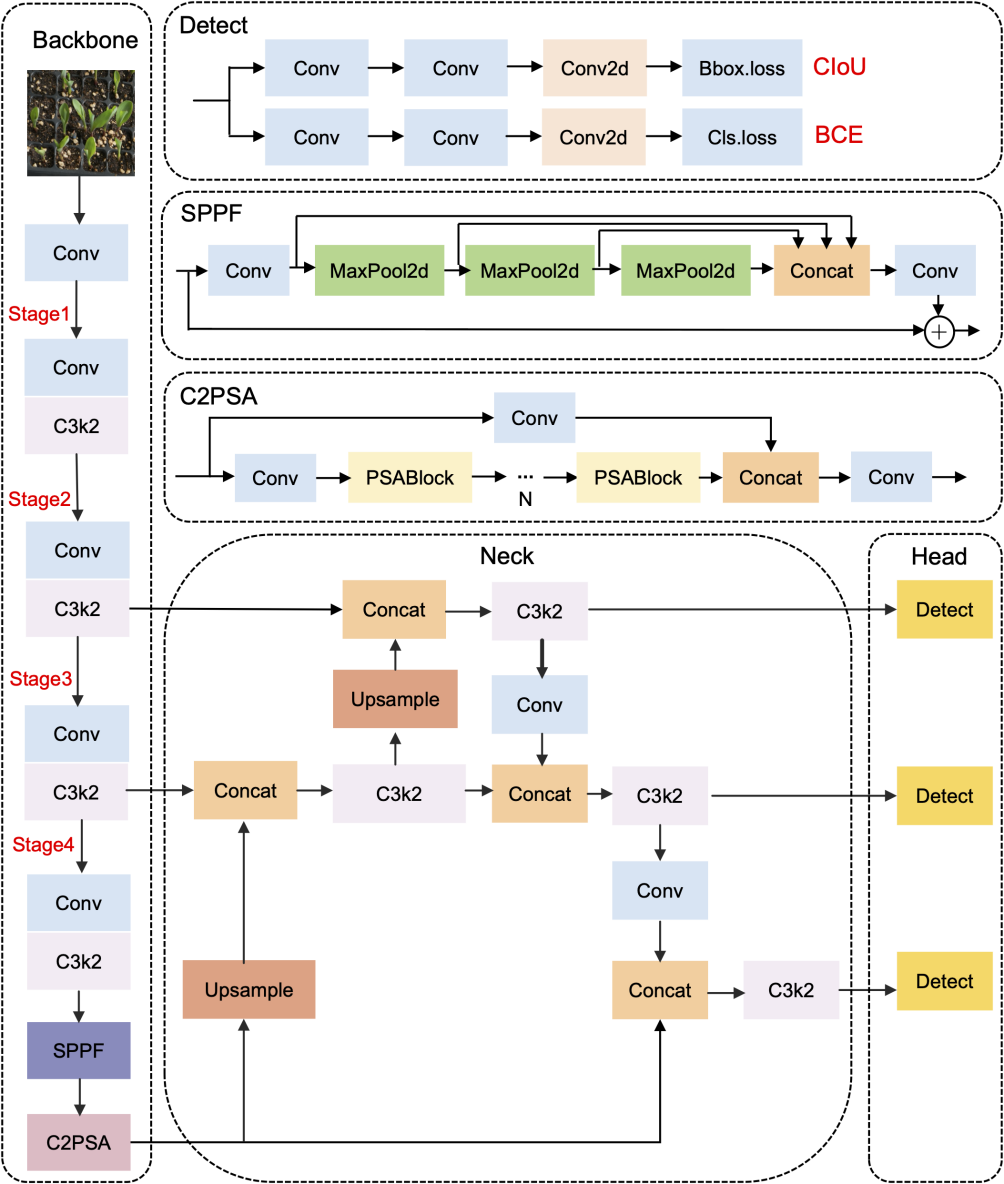
Network architecture of YOLO26n.

The YOLO26n network architecture mainly consists of three components: the backbone, neck, and detection head (**Figure 5**). The backbone is responsible for extracting multiscale empty-cell features from the input images, whereas the neck network fuses features from different hierarchical levels to enhance the representation of target regions. The detection head predicts the bounding box coordinates and class probabilities of empty-cell targets.

#### 2.3.2 Architecture of the proposed MPE-YOLO

**Figure 6:**
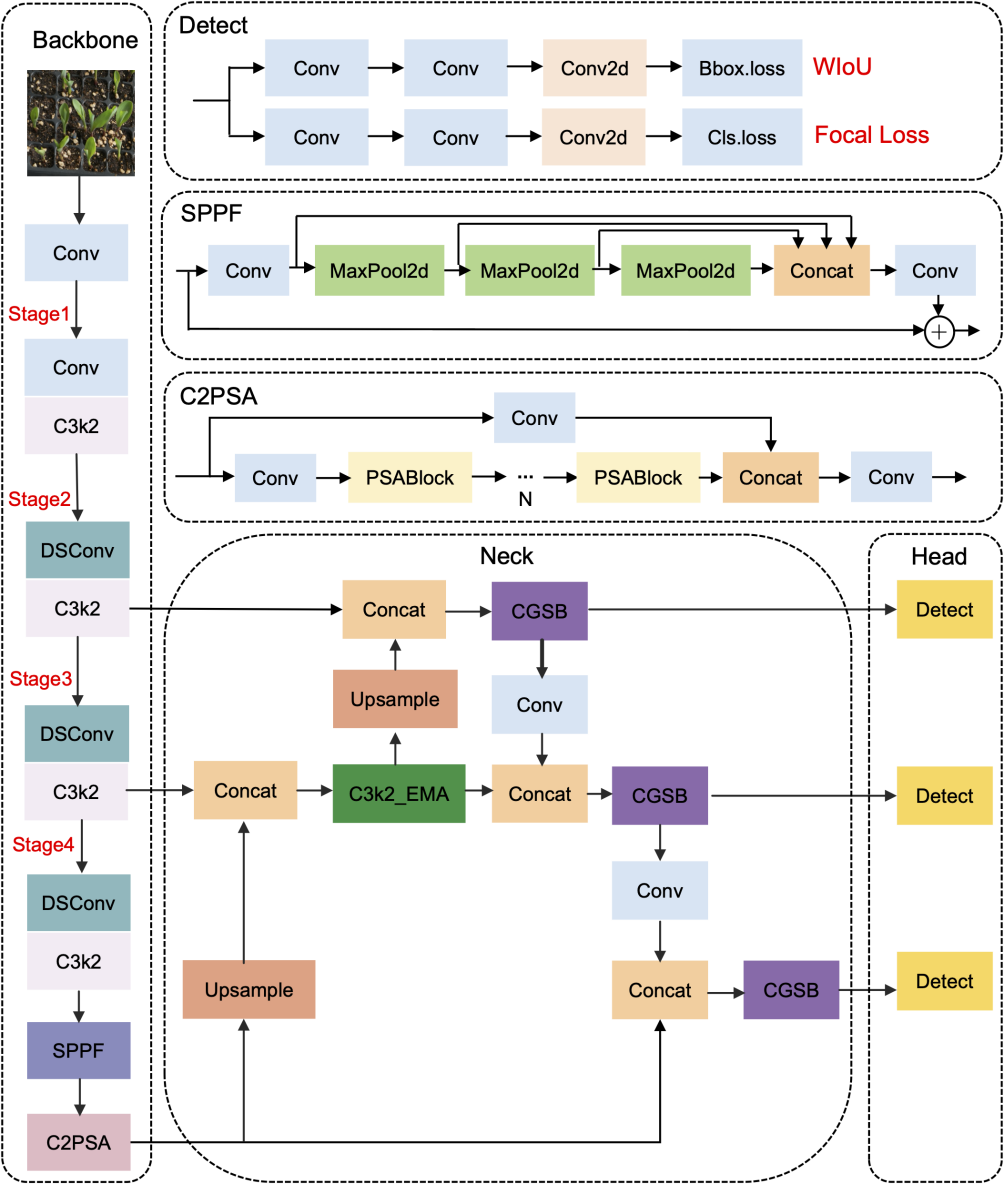
Network architecture of MPE-YOLO.

To improve the robustness of maize plug-tray empty-cell detection against neighboring-leaf occlusion and substrate coverage, we developed MPE-YOLO (**Figure 6**) based on the YOLO26n baseline. While retaining an overall lightweight framework, MPE-YOLO introduces task-specific architectural modifications in feature extraction, fusion, and loss optimization to enhance empty-cell recognition under complex greenhouse conditions. Specifically, DSConv modules were incorporated into selected convolutional layers to reduce the number of model parameters and computational cost, while preserving local empty-cell feature extraction within a lightweight architecture. Subsequently, CGSB modules were introduced into the neck network to strengthen feature interactions and fusion across different hierarchical levels, thereby improving the representation of regions affected by background interference. In addition, a C3k2-EMA module was embedded during deep feature fusion to recalibrate features in key regions, suppress irrelevant activation from complex backgrounds, and enhance feature responses associated with true empty-cell areas. Finally, a dual-focus loss function was designed to further optimize the classification and localization learning for complex samples, thereby improving the discriminative capacity of the model for ambiguous empty-cell targets. These modifications enhance the feature representation and discriminative capacity of MPE-YOLO for empty-cell targets under complex greenhouse conditions without substantially increasing model complexity.

#### 2.3.3 DSConv-based lightweight feature extraction

**Figure 7:**
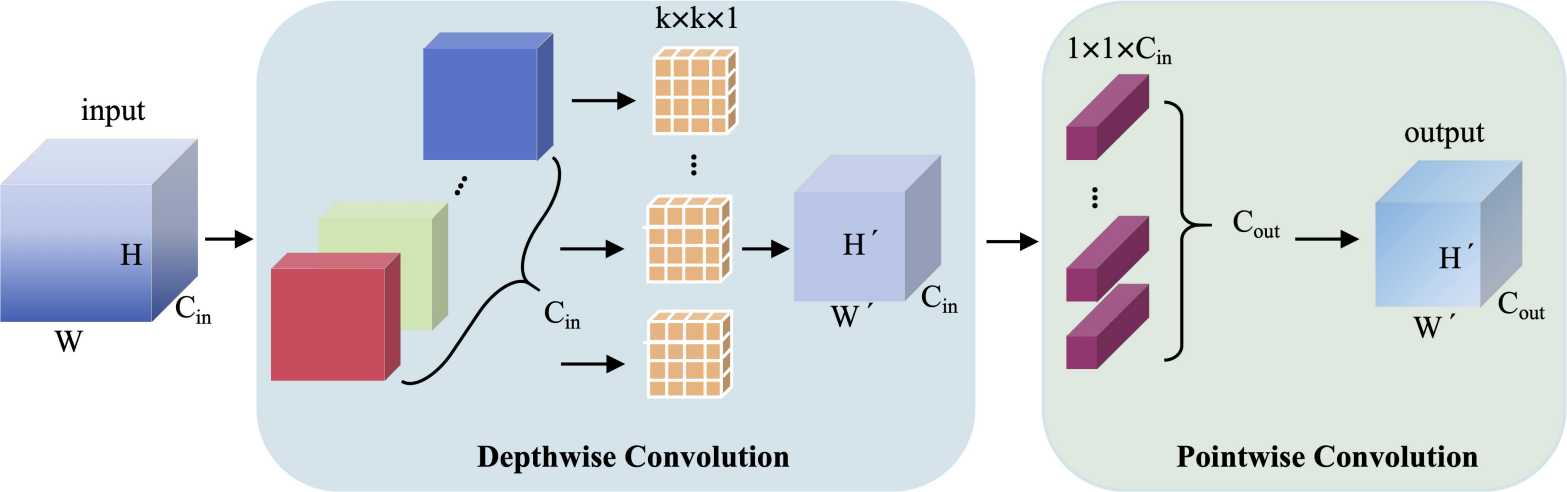
Structural illustration of DSConv.

Depthwise separable convolution, termed DSConv, is a widely used lightweight convolutional structure that reduces the number of model parameters and computational cost while preserving feature extraction capability (Chollet, 2017; Howard et al., 2017; Chen et al., 2022). DSConv decomposes a standard convolution into two sequential operations: depthwise and pointwise convolutions. Depthwise convolution performs spatial convolution independently on each input channel to extract intrachannel local features, whereas pointwise convolution uses a 1×1 convolution to integrate information across channels (**Figure 7**).

Assuming that the input feature map has a spatial size of *H*×*W*, with *C*_in_ input channels, *C*_out_ output channels, and a convolution kernel size of *K*×*K*, the number of parameters and floating-point operations for standard convolution and DSConv can be expressed by **Equations 1**–**4**, respectively. Accordingly, the parameter compression ratio and computational compression ratio of DSConv relative to the standard convolution are both given by 1/*C*_out_ + 1/*K*^2^. This indicates that DSConv can significantly reduce the number of network parameters and floating-point operations, particularly when the number of output channels is high.

(1) $$P_{\text{std}}=K^{2}C_{\text{in}}C_{\text{out}}$$

(2) $$F_{\text{std}}=HWC_{\text{in}}C_{\text{out}}K^{2}$$

(3) $$P_{\text{DSC}}=K^{2}C_{\text{in}}+C_{\text{in}}C_{\text{out}}$$

(4) $$F_{\text{DSC}}=HW(K^{2}C_{\text{in}}+C_{\text{in}}C_{\text{out}})$$

Based on these characteristics, DSConv was incorporated into the lightweight design of the proposed model by replacing standard convolutional layers except for the first two convolutional layers. This configuration reduces the overall network complexity while retaining low-level detailed feature extraction, thereby improving the suitability of the model for edge-device deployment.

#### 2.3.4 CGSB-based lightweight feature fusion

**Figure 8:**
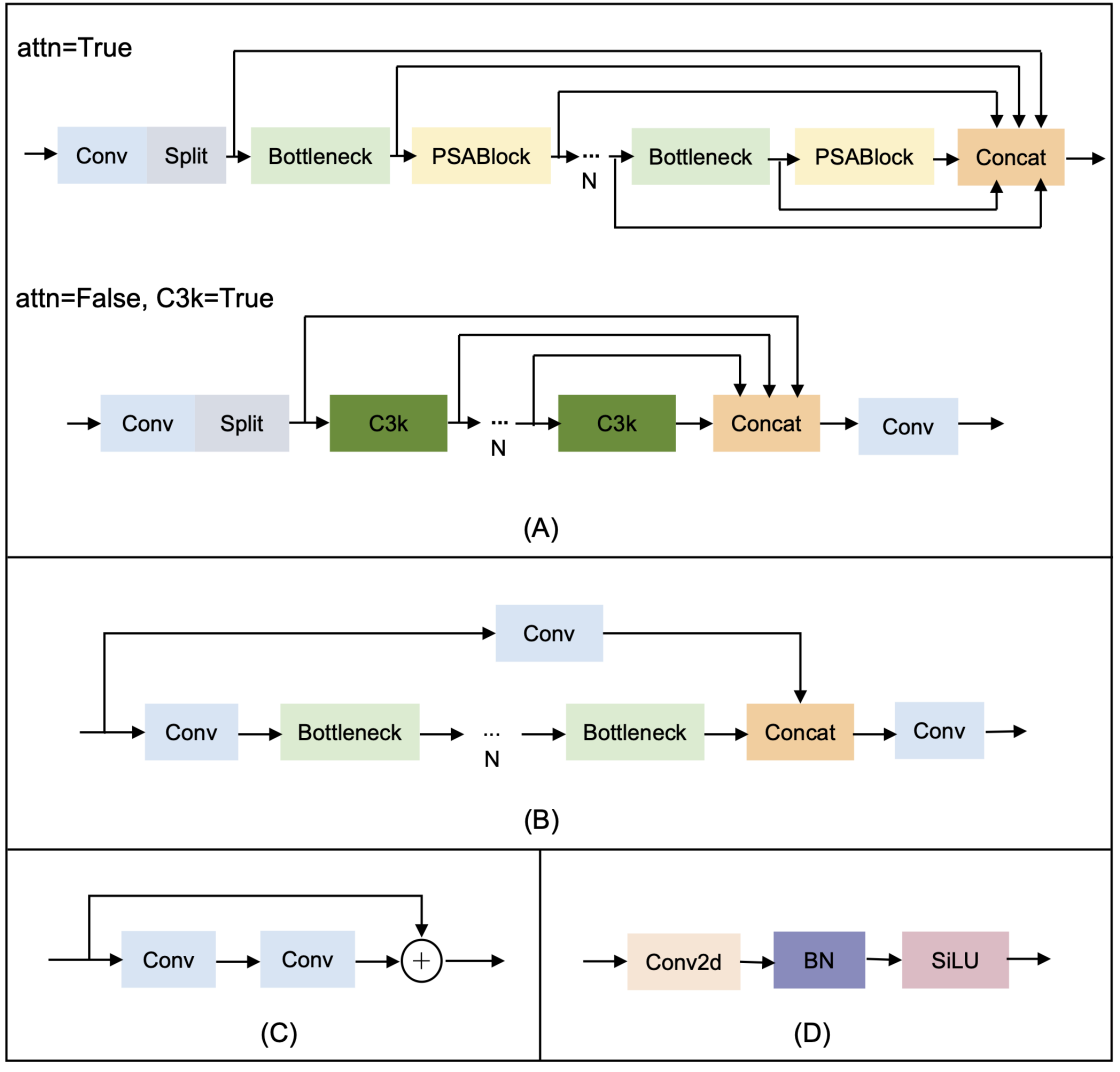
Structural illustrations of **(A)** the C3k2 module, **(B)** the C3k module, **(C)** the Bottleneck module, **(D)** the Conv module.

In the neck of YOLO26n, the C3k2 module (**Figure 8A**) performs feature fusion by combining local feature partitioning with cascaded feature extraction. Its main branch can be configured either as a combination of Bottleneck and PSABlock units or as stacked C3k units, depending on the attention setting. Furthermore, the C3k module (**Figure 8B**) consists of serially connected bottleneck units (**Figure 8C**) and a bypass branch for feature aggregation. Each bottleneck unit contains two convolutional (**Figure 8D**) layers and an optional residual connection. This structure provides effective feature representation, its dependence on standard convolutions leads to a relatively large number of parameters and high computational cost during neck-stage feature fusion, thereby limiting its suitability for lightweight deployment.

**Figure 9:**
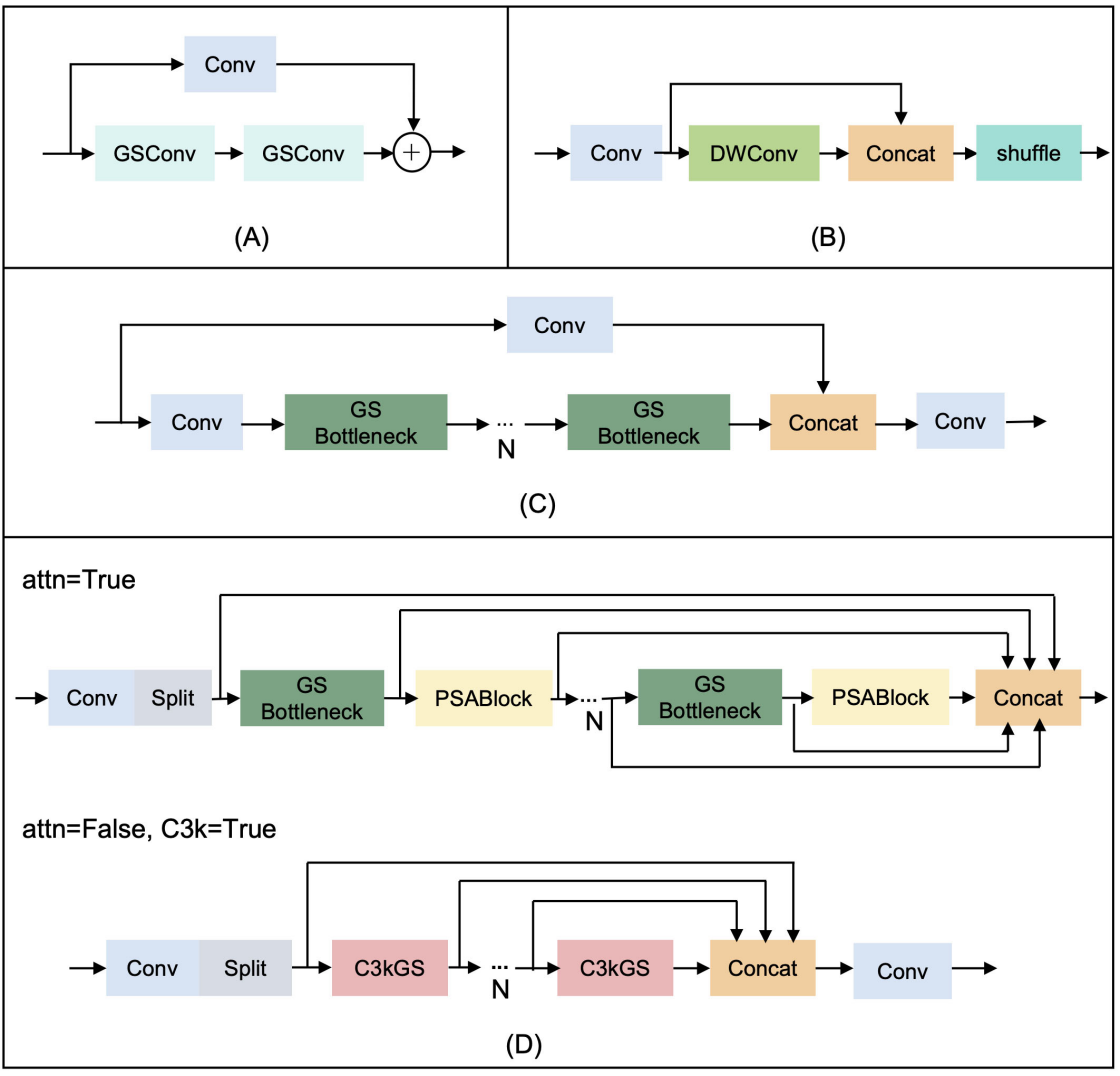
Structural illustrations of **(A)** the GSBottleneck module, **(B)** the GSConv module, **(C)** the C3kGS module, **(D)** the CGSB module.

The ghost–shuffle bottlenecks (GSBottlenecks; **Figure 9A**) is composed of GSConv (**Figure 9B**) modules, which integrate standard convolution, depthwise convolution, feature concatenation and channel shuffle to reduce computational cost while enhancing cross-channel information interaction (Li et al., 2024a). To reduce the complexity of neck-stage feature fusion, this study redesigned the internal structure of C3k2 while retaining its outer local-connection framework. By stacking GSBottlenecks, an internal C3kGS structure (**Figure 9C**) was constructed, which was further incorporated into the lightweight feature-fusion module CGSB (**Figure 9D**). Rather than replacing all the C3k2 modules in the neck network, a detection-head-oriented partial replacement strategy was adopted, in which only the C3k2 modules directly preceding the P3, P4, and P5 detection heads were replaced with CGSB. This design reduces the number of parameters and the computational cost of the neck network while maintaining multiscale feature fusion capability, thereby improving the empty-cell feature representation under complex greenhouse conditions and achieving a balance between model compactness and detection performance.

#### 2.3.5 C3k2-EMA feature recalibration

**Figure 10:**
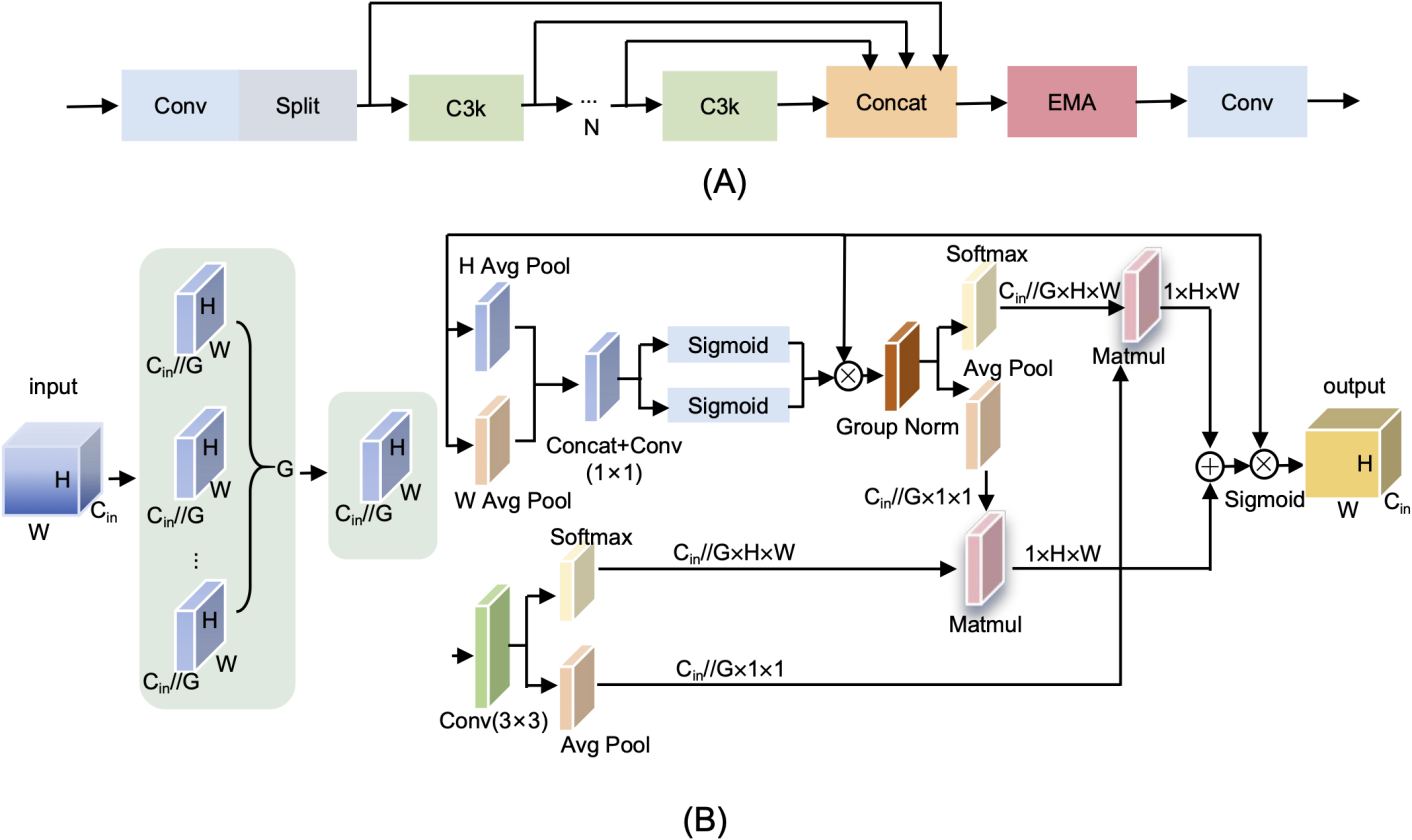
Structural illustrations of **(A)** the C3k2-EMA module, **(B)** the EMA module.

In high-density greenhouse plug-tray scenes, the initial fusion stage of the top-down pathway in the neck network is strongly affected by background interference. When high-level features encoding global empty-cell cues are concatenated with mid-level features containing abundant background details such as leaf edges and substrate textures, feature aliasing may occur. To suppress background-induced feature aliasing, an EMA-based feature recalibration mechanism was introduced at the initial fusion position of the top-down pathway, followed by C3k2-based feature aggregation, thereby forming the C3k2-EMA module (**Figure 10A**).

The EMA mechanism (Ouyang et al., 2023; Li et al., 2024b) models channel-wise and spatial correlations with limited computational overhead and adaptively reweights the fused features (**Figure 10B**). Through feature recalibration, C3k2-EMA enhances responses from true empty-cell regions while suppressing non-target activations caused by substrate covering along plug-cell edges and neighboring-leaf occlusion, thereby improving feature representation in cluttered greenhouse scenes. Its placement at the initial top-down fusion stage also helps preserve discriminative target features for subsequent multiscale propagation and reduces the reliance of CGSB on background noise suppression. In this manner, C3k2-EMA complements the CGSB module and improves empty-cell representation without markedly increasing model complexity.

#### 2.3.6 Dual-focusing loss function

In maize plug-tray empty-cell detection, neighboring-leaf occlusion and substrate covering along cell edges often make the empty-cell appearance and boundaries ambiguous, increasing the difficulty of both classification and localization. To address this challenge, Focal Loss (Qian et al., 2025) and Wise-IoU v3 (WIoU v3) (Tong et al., 2023) were integrated to construct a dual-focusing loss function, termed DF-Loss, which enables task-specific optimization of both classification and localization.

In the classification branch, Focal Loss was implemented by applying a focal weighting term to the binary cross-entropy loss with logits. Specifically, the baseline classification loss was first formulated as shown in **Equation 5**.

(5) $$L_{\text{BCE}}=BCEWithLogits(p,y)$$

where *p* denotes the predicted logits from the classification branch, and *y* represents the corresponding target class score. Based on *L*_BCE_, the probability-related term used for focal modulation was calculated using **Equation 6**, and the final classification loss was obtained using **Equation 7**.

(6) $$p_{t}=\exp(-L_{\text{BCE}})$$

(7) $$L_{\text{cls}}=\alpha_{t}{\left(1-p_{t}\right)}^{\gamma }L_{\text{BCE}}$$

where *α_t_* is the class-balancing factor, and γ is the focus parameter. It can be inferred that, for correctly and stably classified samples, *L*_BCE_ is small and *p_t_* approaches 1, so the modulation term (1 − *p_t_*)^γ^ suppresses their contribution to the classification loss. Conversely, hard empty-cell samples with weak boundaries produce relatively smaller *p_t_* values and are assigned larger classification loss weights. This strategy guides the classification branch to focus more on hard samples caused by neighbouring-leaf occlusion and substrate coverage along cell edges, thereby improving classification reliability under complex plug-tray conditions.

In the localization branch, WIoU v3 was adopted to replace the default CIoU loss. First, a distance-attention term, *R*_WIOU_, was introduced to characterize the center displacement between the predicted and ground-truth bounding boxes, and was formulated as shown in **Equation 8**.

(8) $$R_{\text{WIoU}}=\exp(\frac{{\left(x-x^{gt}\right)}^{2}+{\left(y-y^{gt}\right)}^{2}}{{\left(W_{g}^{2}+H_{g}^{2}\right)}^{*}})$$

where (*x,y*) and (*x*^gt^,*y*^gt^)denote the center coordinates of the predicted and ground-truth bounding boxes, respectively. *W*_g_ and *H*_g_ represent the width and height of the smallest enclosing box that covers both the predicted and ground-truth boxes, respectively. The symbol * indicates that this term does not participate in gradient updates during backpropagation. Furthermore, WIoU v3 introduces the sample outlier degree *β*, which quantifies the deviation of the current sample from the training distribution, as formulated in **Equation 9**.

(9) $$\beta =\frac{L_{\text{IoU}}^{*}}{{\bar{L}}_{\text{IoU}}}$$

where $L_{IoU}^{*}$ denotes the IoU loss of the current sample, and ${\bar{L}}_{IoU}$ represents the average historical loss. Based on **Equation 9**, the non-monotonic focusing coefficient γ was constructed as shown in **Equation 10**, and the final regression loss was formulated as shown in **Equation 11**.

(10) $$\gamma =\frac{\beta }{\delta \alpha^{\beta -\delta }}$$

(11) $$L_{\text{reg}}=\gamma \cdot R_{\text{WIoU}}(1-\text{IoU})$$

where *α* and *δ* are the hyperparameters that control the shape of the focusing function. This mechanism adaptively adjusts the regression gain according to sample quality, suppressing harmful gradients from low-quality samples with severe occlusion or ambiguous boundaries, thereby improving localization robustness under complex backgrounds.

Based on the above design, the proposed DF-Loss was defined by combining the focal classification loss and the WIoU v3 regression loss, as formulated in **Equation 12**.

(12) $$L_{DF}=\lambda_{cls}L_{cls}+\lambda_{reg}L_{reg}$$

where *L*_cls_ denotes the Focal Loss, *L*_reg_ represents the WIoU v3 regression loss. λ_cls_ and λ_reg_ denote the weighting coefficients of the corresponding loss branches.

By introducing sample reweighting into both the classification and localization branches, DF-Loss increases the optimization emphasis on hard empty-cell samples and informative localization samples, thereby improving the accuracy of the model in detecting empty-cell targets under complex greenhouse conditions.

#### 2.3.7 iOS-based deployment of MPE-YOLO

To enable on-site empty-cell detection in maize plug trays, the proposed MPE-YOLO model was deployed on an iPhone, and an iOS application named *Corn Empty-Cell Detector* was developed. The deployment was implemented using Core ML, Apple’s machine-learning framework for local inference on iOS devices. Specifically, the trained.pt weight file of MPE-YOLO was converted into Core ML.mlpackage format using the model export function provided by the Ultralytics framework, and then integrated into the mobile application for local inference. During model conversion, the key parameters, namely format and image size, were set to coreml and 640, respectively, ensuring consistency with the input size used during model training and evaluation.

Compared with cloud-based deployment, local iOS deployment reduces the dependence on network connectivity, avoids unnecessary image data transmission, and shortens the feedback time during on-site inspection. The lightweight design of MPE-YOLO further lowers the computational burden of mobile inference, improving its suitability for resource-constrained devices. Overall, this deployment strategy provides a practical implementation pathway for rapid empty-cell detection in greenhouse maize plug-tray seedling production.

## 3 Experimental results

### 3.1 Experimental environment and training parameters

In this study, all experiments were conducted in the same software and hardware environment to ensure fair and reliable comparisons among the different models and improvement strategies. The detailed configurations are listed in **Table 3**. During model training, the input image size was set to 640, the batch size to 16, and the maximum number of epochs to 200. Mosaic augmentation was disabled during the last 30 epochs by setting the value of close_mosaic to 30, allowing the model to fine-tune on more realistic image distributions before training termination. The model parameters were updated using the AdamW optimizer, and the learning rate was dynamically adjusted using a cosine annealing strategy. To improve reproducibility and evaluate model stability, repeated experiments were conducted using five random seeds, namely 0, 15, 150, 999, and 1500. For each seed, the model was independently trained under the same experimental settings, and deterministic training was performed. Except for the key hyperparameters described above, all other training settings were maintained at the default values of the corresponding version of the training framework.

**Table 3:** Experimental environment configuration.

Environment	Configuration
Operating System	Windows11
CPU	Intel Core i7-14700KF
GPU	NVIDIA GeForce RTX 4080
Display memory	16GB
RAM	32GB
Programming language	Python 3.11
Deep Learning Framework	torch 2.7.0+cu118

### 3.2 Evaluation metrics

To comprehensively evaluate model performance, precision (P), recall (R), mAP@0.5, mAP@0.5:0.95, parameters (Params), giga floating-point operations (GFLOPs), and frames per second (FPS) were used as evaluation metrics. The precision and recall were used to assess the ability of the model to control false and missed detections, respectively. mAP@0.5 reflects the overall detection performance at an IoU threshold of 0.5, while mAP@0.5:0.95 represents the mean average precision averaged across IoU thresholds ranging from 0.5 to 0.95. Model efficiency was evaluated using Params, GFLOPs, and FPS, which represent model size, theoretical computational complexity, and practical inference speed, respectively. Given that this study aims to balance empty-cell detection accuracy under complex greenhouse plug-tray backgrounds with the efficiency required for mobile deployment, model selection jointly considers detection performance, localization quality, model complexity, and inference speed, rather than relying on a single evaluation metric. For repeated experiments, the results of ablation experiments, model comparison experiments, stratified evaluations, and empty-cell-free false detection analysis are presented as the mean ± standard deviation across five repeated experiments with different random seeds. The PR curves, training loss curves, representative detection visualizations, and edge-device deployment tests were generated using the model trained with seed = 15.

### 3.3 Ablation experiment

To evaluate the contribution of each improved module to maize plug-tray empty-cell detection, ablation experiments were conducted using YOLO26n as a baseline model. Single-module replacement and multimodule combination strategies were used to assess the effects of DSConv, CGSB, C3k2-EMA and DF-Loss on the detection performance and model complexity. Precision, mAP@0.5, Params, GFLOPs, and FPS were used as the main evaluation metrics.

As shown in **Table 4**, introducing DSConv alone substantially improved inference efficiency, although it led to a slight decrease in precision compared with the baseline. This indicates that DSConv is mainly effective for lightweight feature extraction, but using it alone may weaken detection stability. In contrast, introducing CGSB alone maintained detection accuracy while reducing model complexity. When DSConv and CGSB were used together, the model achieved a more favorable balance between compactness and detection performance than using either module alone. These results demonstrate that DSConv and CGSB are compatible and can be jointly used for lightweight model design.

**Table 4:** Ablation results of the proposed modules.

Model	DSConv	CGSB	C3k2-EMA	DF-Loss	P/%	mAP@0.5/%	Params/M	GFLOPs	FPS
1					92.2 ± 0.5	96.7 ± 0.2	2.38	5.2	495.0 ± 32.2
2	✓				90.7 ± 1.1	96.7 ± 0.4	1.79	3.9	550.7 ± 39.6
3		✓			92.2 ± 0.7	96.9 ± 0.4	2.09	4.8	498.2 ± 35.8
4	✓	✓			93.0 ± 1.0	96.8 ± 0.6	**1.50**	**3.5**	**579.1 ± 30.2**
5	✓	✓	✓		93.3 ± 0.8	96.8 ± 0.2	1.51	3.6	570.7 ± 39.6
6	✓	✓	✓	✓	**93.8 ± 0.5**	**97.0 ± 0.1**	1.51	3.6	574.3 ± 25.4

Bold values indicate the best-performing results.

Based on this lightweight structure, C3k2-EMA was further introduced to enhance feature representation in complex plug-tray scenes. With only a negligible increase in model size, C3k2-EMA improved precision by 0.3 percentage points while maintaining the same mAP@0.5, suggesting that attention-based feature recalibration may help the model better distinguish empty cells from neighboring-leaf occlusion and substrate coverage. After DF-Loss was added, the final model achieved the best overall detection performance while maintaining a lightweight architecture and high inference efficiency.

Overall, the ablation results demonstrate that each proposed component contributed to the final performance from different perspectives. DSConv and CGSB effectively improved lightweight efficiency, whereas C3k2-EMA and DF-Loss further enhanced detection accuracy. These modules showed good complementarity, enabling MPE-YOLO to achieve a better balance among detection accuracy, model complexity, and inference efficiency.

### 3.4 Comparison of different detection models

**Figure 11:**
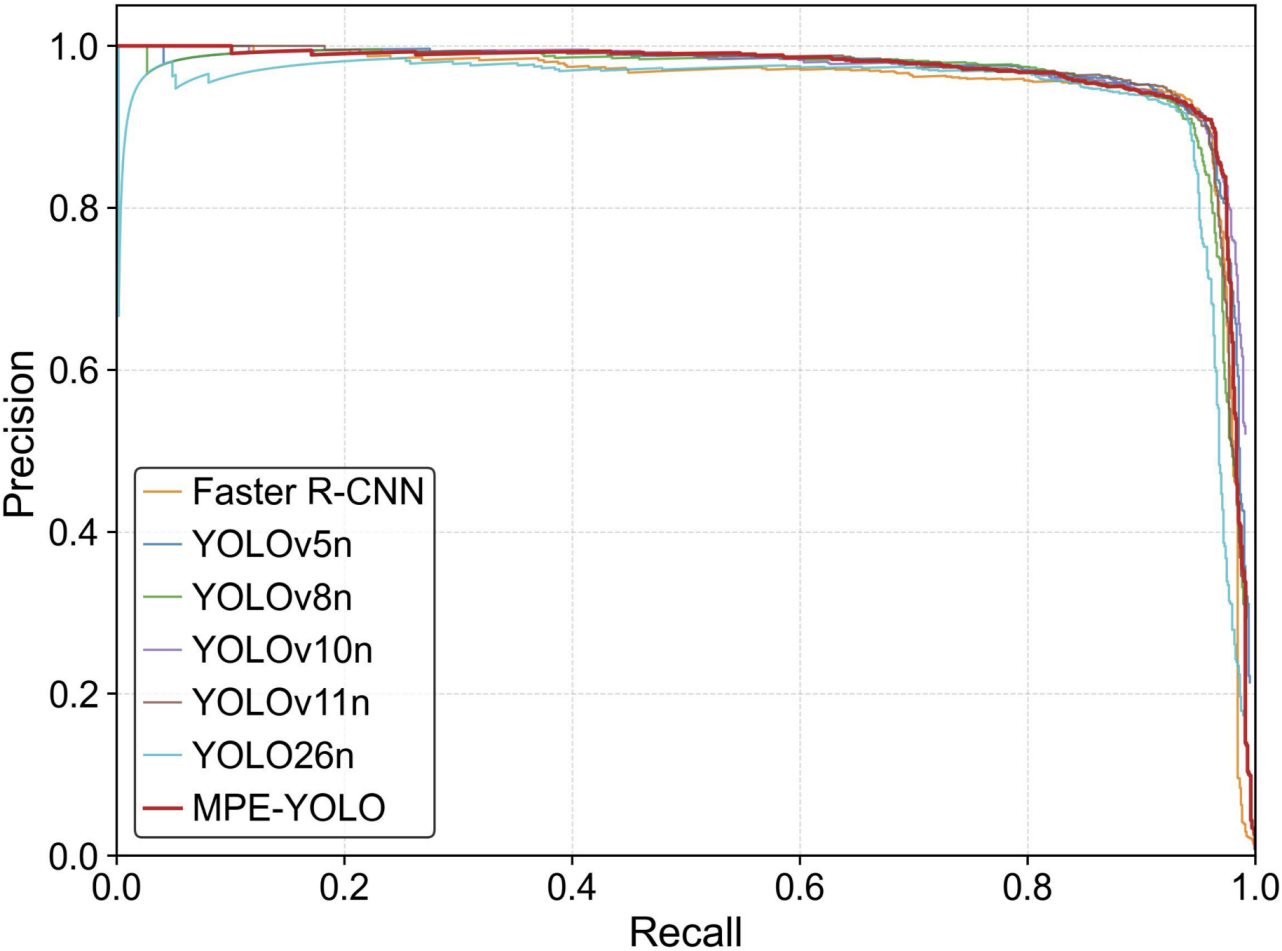
Precision-recall curves of different detection models.

The effectiveness of the proposed model for maize plug-tray empty-cell detection was further evaluated by comparing it with Faster R-CNN, YOLOv5n, YOLOv8n, YOLOv10n, YOLOv11n, and YOLO26n. All models were trained and evaluated using the same dataset, training strategy, and experimental environment. **Figure 11** shows the PR curves of all models, and **Table 5** presents the corresponding quantitative results.

**Table 5:** Performance comparison of different deep learning models on test dataset.

Model	P/%	R/%	mAP@0.5/%	mAP@0.5:0.95/%	Params/M	GFLOPs	FPS
Faster R-CNN	92.6 ± 0.3	93.7 ± 0.8	95.0 ± 0.3	82.4 ± 0.4	41.76	134.3	63.9 ± 5.6
YOLOv5n	91.1 ± 0.8	**94.5 ± 0.8**	96.8 ± 0.1	84.6 ± 0.1	2.50	7.1	369.0 ± 29.9
YOLOv8n	91.7 ± 1.1	92.9 ± 1.2	96.5 ± 0.3	84.8 ± 0.4	3.01	8.1	393.4 ± 8.7
YOLOv10n	92.3 ± 1.1	93.6 ± 0.9	96.5 ± 0.3	85.9 ± 0.2	2.27	6.5	481.2 ± 66.5
YOLOv11n	91.7 ± 0.8	93.8 ± 0.6	96.9 ± 0.2	86.1 ± 0.4	2.58	6.3	365.5 ± 19.9
YOLO26n	92.2 ± 0.5	93.8 ± 0.4	96.7 ± 0.2	86.5 ± 0.2	2.38	5.2	495.0 ± 32.2
MPE-YOLO	**93.8 ± 0.5**	94.0 ± 0.4	**97.0 ± 0.1**	**86.6 ± 0.3**	**1.51**	**3.6**	**574.3 ± 25.4**

Bold values indicate the best-performing results.

As shown in **Figure 11**, all compared models achieved high precision and recall values in maize plug-tray empty-cell detection. Among them, MPE-YOLO maintained higher precision over a wide recall range, particularly in the high-recall region where false positives are more likely to occur. This indicates that the proposed model can improve the reliability of empty-cell detection while effectively suppressing false detections.

As listed in **Table 5**, Faster R-CNN achieved relatively high precision and recall. However, its mAP@0.5 and mAP@0.5:0.95 values were lower than those of the lightweight YOLO models. In addition, Faster R-CNN had 41.76 M parameters and 134.3 GFLOPs, indicating a substantially larger model size and higher computational cost, which may limit its suitability for deployment on edge devices. Among the lightweight YOLO models, YOLOv5n, YOLOv8n, YOLOv10n, YOLOv11n, and YOLO26n all achieved competitive detection performance. Nevertheless, compared with MPE-YOLO, these models generally showed a less favorable trade-off among detection accuracy, model complexity, and inference speed.

Collectively, MPE-YOLO achieved the highest mean precision, mAP@0.5, and mAP@0.5:0.95 among all comparison models, while maintaining the lowest model complexity and the highest mean inference speed. The relatively small standard deviations of the main accuracy metrics further indicate that the proposed model produced stable detection performance across repeated experiments. Although FPS showed relatively larger variation, MPE-YOLO still achieved the highest mean inference speed. These results demonstrate that MPE-YOLO provides a better balance between detection accuracy, lightweight architecture, and inference efficiency, making it more suitable for real-time maize plug-tray empty-cell detection.

### 3.5 Performance under complex background conditions and false detection analysis

#### 3.5.1 Performance under occlusion and substrate-covering conditions

The robustness of the proposed model under different background conditions was further evaluated by dividing the original test set into five background-condition-specific subsets, including simple scenes, slight neighboring-leaf occlusion, slight substrate covering of cell edges, severe neighboring-leaf occlusion, and severe substrate covering of cell edges. All models were trained using the same training set, and only the test images were regrouped according to the dominant background interference for separate evaluation.

As shown in **Table 6**, both YOLO26n and MPE-YOLO achieved high detection accuracy under simple scenes, indicating that empty cells can be reliably detected when the plug-tray background is clear and interference is limited. Under slight occlusion and slight substrate covering conditions, MPE-YOLO generally achieved higher mean detection performance than YOLO26n, especially in terms of mAP. For the more challenging severe occlusion and severe substrate covering scenarios, the detection performance of both models decreased compared with simple scenes. However, MPE-YOLO still achieved higher mean precision, recall, and mAP values than YOLO26n in most metrics.

**Table 6:** Performance comparison of YOLO26n and MPE-YOLO under neighboring-leaf occlusion and substrate-covering conditions.

Condition	Images	Targets	Model	P/%	R/%	mAP@0.5/%	mAP@0.5:0.95/%
Simple scene	51	157	YOLO26n	96.8 ± 0.5	96.4 ± 0.4	98.3 ± 0.3	**88.3 ± 0.9**
			MPE-YOLO	**96.9 ± 0.3**	**96.6 ± 0.8**	**98.4 ± 0.5**	88.2 ± 0.7
Slight occlusion	54	178	YOLO26n	95.6 ± 0.6	94.3 ± 1.1	97.3 ± 0.4	84.9 ± 0.1
			MPE-YOLO	**95.7 ± 0.6**	**95.3 ± 0.6**	**97.7 ± 0.2**	**86.8 ± 0.4**
Slight substrate covering	43	131	YOLO26n	93.5 ± 0.2	**96.3 ± 1.0**	97.1 ± 0.4	87.9 ± 0.3
			MPE-YOLO	**94.4 ± 0.4**	96.2 ± 0.7	**97.5 ± 0.1**	**88.7 ± 0.2**
Severe occlusion	25	64	YOLO26n	87.3 ± 2.2	87.9 ± 0.6	93.3 ± 0.9	81.8 ± 0.7
			MPE-YOLO	**88.6 ± 0.8**	**89.8 ± 1.1**	**94.0 ± 0.5**	**82.1 ± 0.6**
Severe substrate covering	16	56	YOLO26n	93.4 ± 1.2	92.3 ± 1.5	95.9 ± 0.8	87.0 ± 0.5
			MPE-YOLO	**94.4 ± 0.8**	**94.0 ± 1.3**	**96.3 ± 0.3**	**87.6 ± 0.7**

Bold values indicate the best-performing results.

These results suggest that MPE-YOLO maintained competitive performance in simple scenes and showed better robustness than YOLO26n under occlusion and substrate-covering conditions. Although the number of images and targets varied among the five subsets, the results indicate that MPE-YOLO has stronger adaptability to the complex greenhouse plug-tray backgrounds commonly encountered in practical empty-cell detection.

#### 3.5.2 False detection analysis in empty-cell-free scenes

An additional empty-cell-free test set was constructed to evaluate whether the model produced false empty-cell detections under complex greenhouse background conditions when no empty-cell targets were present. Specifically, 30 complete plug-tray images without empty cells were selected from the original captured data. Following the same preprocessing procedure described above, each complete plug tray was cropped into eight 4×4 patches, resulting in a total of 240 empty-cell-free 4×4 patches. This additional dataset was used only for false detection analysis in empty-cell-free scenarios and was not involved in model training, validation, or model selection.

For patch-level analysis, a 4×4 patch was regarded as a false-positive patch if at least one empty-cell bounding box was predicted in that patch. For tray-level analysis, the detection results of the eight 4×4 patches cropped from the same complete plug tray were regrouped according to their original tray. The number of false detections in each tray was obtained by summing all predicted empty-cell bounding boxes in its eight patches. False detections in empty-cell-free scenes were evaluated using the following three metrics, as defined in **Equations 13**–**15**.

(13) $${\text{FPR}}_{\text{patch}}=\frac{N_{\text{fp}}}{N_{\text{patch}}}$$

(14) $${\text{FD}}_{\text{tray}}=\frac{B_{\text{fp}}}{N_{\text{tray}}}$$

(15) $${\text{FPR}}_{\text{tray}}=\frac{N_{\text{ft}}}{N_{\text{tray}}}$$

where FPR_patch_ denotes the patch-level false positive rate, *N*_fp_ denotes the number of empty-cell-free patches with at least one false detection, and *N*_patch_ denotes the total number of empty-cell-free patches. FD_tray_ denotes the average number of false detections per tray, *B*_fp_ denotes the total number of false empty-cell bounding boxes in all empty-cell-free patches, and *N*_tray_ denotes the number of complete empty-cell-free trays. FPR_tray_ denotes the tray-level false positive rate, and *N*_ft_ denotes the number of trays with at least one false detection.

As shown in **Table 7**, MPE-YOLO produced fewer false detections than YOLO26n at both the patch and tray levels. For the patch-level analysis, MPE-YOLO reduced the false positive rate from 23.7% to 12.9%, indicating a lower tendency to misidentify normal plug cells as empty cells in local 4×4 patches. After regrouping the patch-level predictions into complete trays, MPE-YOLO also reduced the average number of false detections per tray. Meanwhile, the tray-level false positive rate decreased from 32.5% to 13.2%, suggesting that fewer complete trays contained false empty-cell detections. These results indicate that MPE-YOLO improved detection reliability by reducing false detections in empty-cell-free scenes.

**Table 7:** Patch-level and tray-level false detection results in empty-cell-free scenes.

Model	FPR_patch_/%	FD_tray_	FPR_tray_/%
YOLO26n	23.7 ± 6.6	3.1 ± 0.9	32.5 ± 10.0
MPE-YOLO	**12.9 ± 4.7**	**1.3 ± 0.5**	**13.2 ± 4.9**

Bold values indicate the best-performing results.

### 3.6 Training loss convergence

To compare the training convergence behavior of the baseline YOLO26n and the improved MPE-YOLO, the classification and localization loss curves of the two models were analyzed.

**Figure 12:**
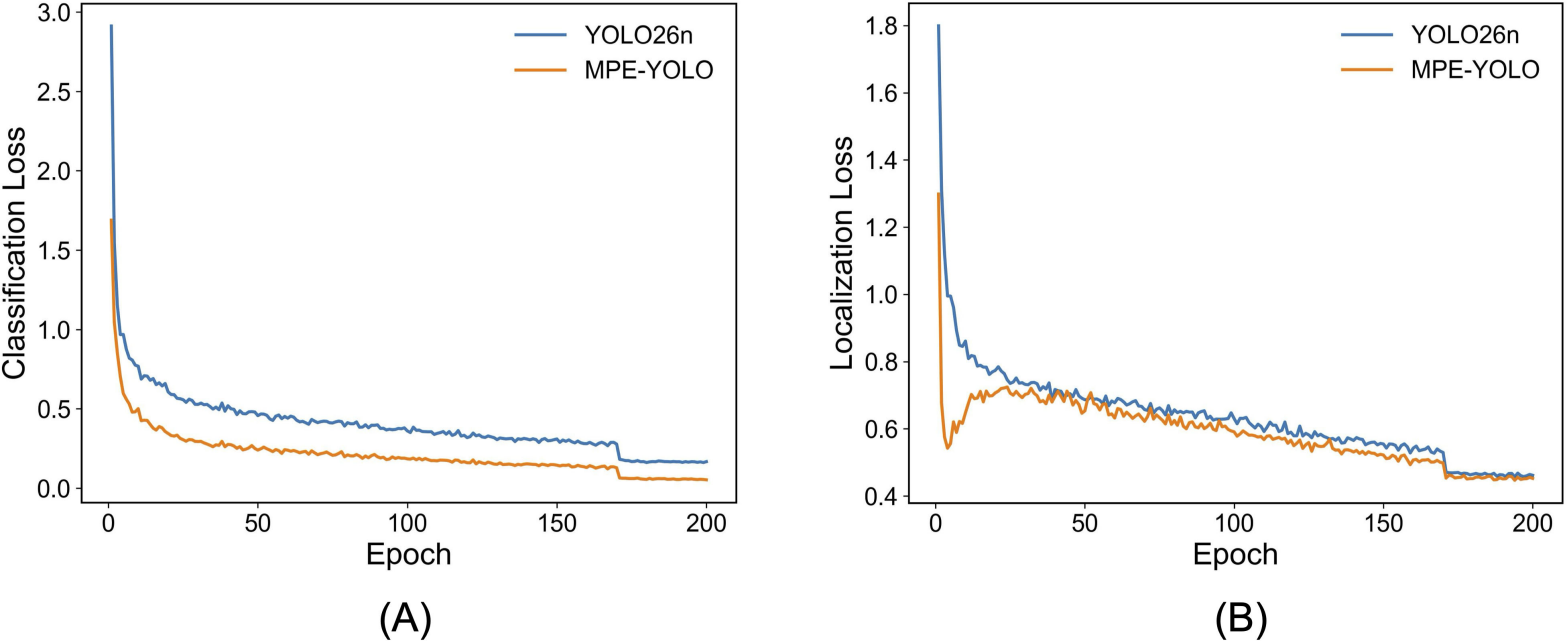
Loss function curves of YOLO26n and MPE-YOLO. **(A)** Classification loss curve. **(B)** Localization loss curve.

As shown in **Figure 12A**, the classification loss of MPE-YOLO decreased more rapidly during the early training stage and remained generally lower than that of YOLO26n throughout the training process, eventually converging to a lower level. **Figure 12B** shows that the localization loss of MPE-YOLO declines rapidly at the beginning of training and then gradually stabilizes after a short period of fluctuation. During most of the training stages, its localization loss was lower than that of YOLO26n and finally converged to a lower value. Overall, MPE-YOLO exhibited lower loss levels and better convergence characteristics in both the classification and localization branches, indicating that the improved model facilitates the optimization process during training.

### 3.7 Visualization

#### 3.7.1 Visualization of detection results under complex backgrounds

**Figure 13:**
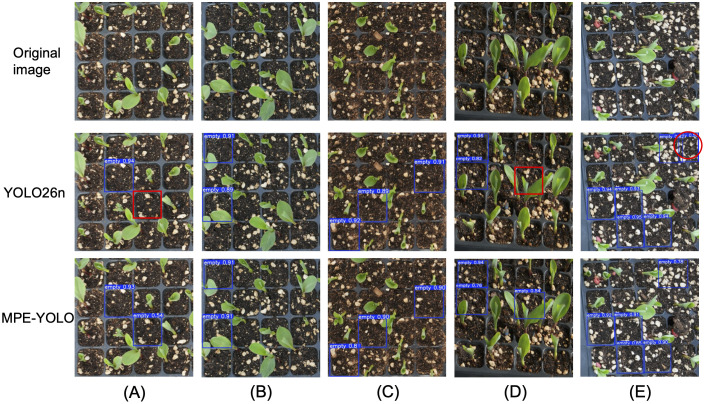
Detection comparison of YOLO26n and MPE-YOLO under different complex background conditions. **(A)** Simple scene. **(B)** Slight occlusion. **(C)** Slight substrate covering. **(D)**Severe occlusion. **(E)** Severe substrate covering. Red squares indicate missed detections, and red circles indicate false detections.

To evaluate the practical detection performance under the complex greenhouse plug-tray backgrounds considered in this study, representative samples from the test set were selected for visualization. Under simple scenes (**Figure 13A**), slight neighboring-leaf occlusion (**Figure 13B**), and slight substrate covering along the plug-cell boundaries (**Figure 13C**), both YOLO26n and MPE-YOLO identified most empty-cell targets, with the predicted bounding boxes showing good agreement with the empty-cell regions. However, YOLO26n produced an individual missed detection, whereas MPE-YOLO successfully detected the corresponding empty-cell target. Under severe occlusion (**Figure 13D**), empty cells in the original image were clearly occluded. YOLO26n failed to detect occluded empty cells, whereas MPE-YOLO detected empty cells. This result indicates that when the visible region of the empty cell was reduced and the boundary information was incomplete, MPE-YOLO retained a better detection capability for occluded empty cells than YOLO26n. Under severe substrate covering (**Figure 13E**), YOLO26n incorrectly detected an incomplete adjacent plug-cell region as an empty cell and produced a smaller bounding box for the true empty cell. In contrast, MPE-YOLO correctly detected the true empty cells affected by severe substrate coverage and did not produce false detections.

MPE-YOLO produces stable detection results for simple, occluded, and substrate-covered scenes. Compared with YOLO26n, MPE-YOLO reduced missed and false detections under severe occlusion and substrate covering, indicating better adaptability to complex background conditions caused by overlapping leaves and substrate-covered cell edges.

#### 3.7.2 HiResCAM-based feature response visualization

**Figure 14:**
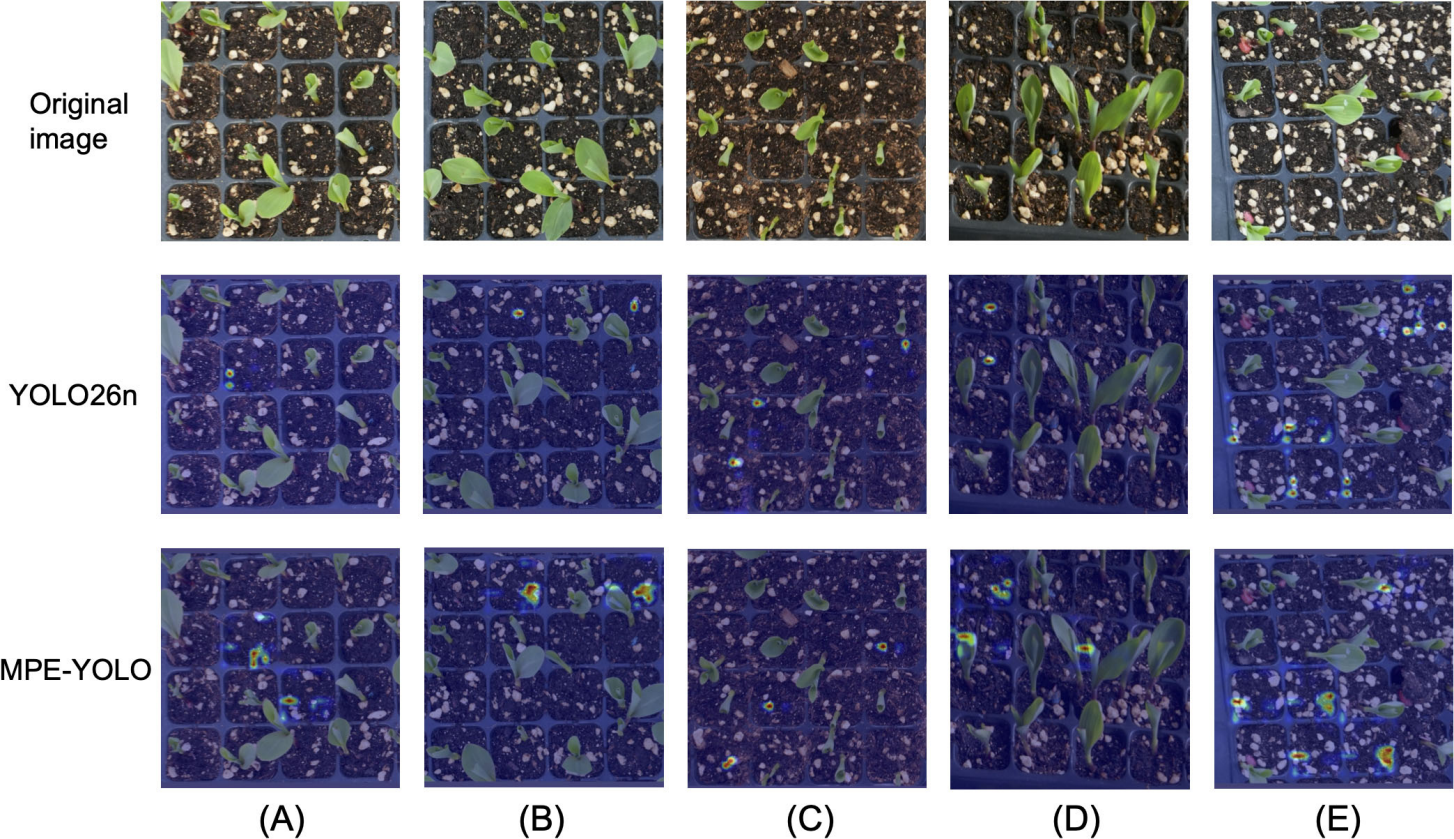
HiResCAM heatmap comparison of YOLO26n and MPE-YOLO under different complex background conditions. **(A)** Simple scene. **(B)** Slight occlusion. **(C)** Slight substrate covering. **(D)** Severe occlusion. **(E)** Severe substrate covering.

To further examine the feature response regions of the models under complex greenhouse plug-tray background conditions, HiResCAM (Pinheiro et al., 2026) was used to visualize the empty-cell detection results for YOLO26n and MPE-YOLO. Both YOLO26n and MPE-YOLO produced responses in empty-cell-related regions under different background conditions, but their response distributions differed. In the simple scene (**Figure 14A**), the response of YOLO26n was primarily concentrated in local regions within the target area, whereas MPE-YOLO generated a more continuous response over the empty-cell region. Under slight occlusion (**Figure 14B**) and substrate covering (**Figure 14C**), the high-response regions of MPE-YOLO exhibited more complete coverage and better spatial agreement with the empty-cell area. Under severe occlusion (**Figure 14D**) and substrate covering (**Figure 14E**), the differences in the feature responses between the two models became more evident. The heat maps of YOLO26n were mainly characterized by several discrete and small high-response regions, whereas MPE-YOLO generally produced stronger and more continuous responses within the target empty-cell regions.

YOLO26n tended to generate localized point-like responses. In comparison, MPE-YOLO produced more continuous target-region responses and maintained more complete coverage of empty-cell areas in the most complex backgrounds. These visualization results were consistent with the detection experiments and further demonstrated the feature-response advantage of MPE-YOLO under complex greenhouse plug-tray backgrounds caused by neighboring-leaf occlusion and substrate-covered cell edges.

### 3.8 Functionality and performance evaluation of the mobile application

**Figure 15:**
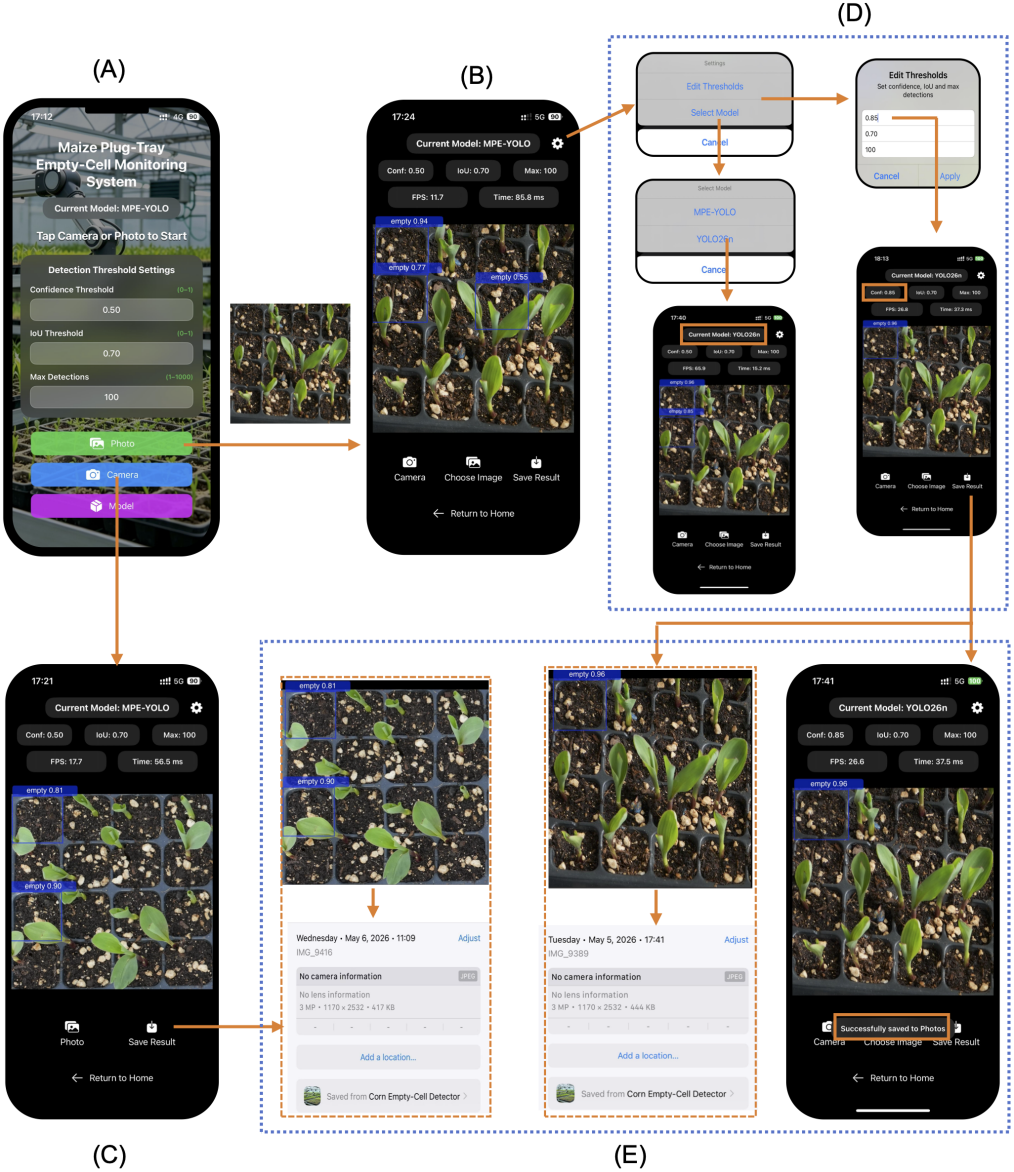
Interfaces of the developed APP. **(A)** Home page. **(B)** Image detection interface. **(C)** Camera detection interface **(D)** Settings interface and corresponding detection result. **(E)** Result saving interface.

The Corn Empty-Cell Detector application mainly consists of functional modules, including the main interface, image-based detection, camera-based detection, parameter settings, and result saving. The main interface (**Figure 15A**) was used to initialize the detection parameters and select the model. The image-based detection interface (**Figure 15B**) supports the import of plug-tray images from a mobile photo album for empty-cell detection, while the camera-based detection interface (**Figure 15C**) enables real-time image acquisition and empty-cell detection using a built-in camera. The parameter setting interface (**Figure 15D**) allows adjustment of the confidence threshold, IoU threshold, and maximum number of detected targets, and supports switching between MPE-YOLO and YOLO26n to meet different requirements for detection sensitivity and model comparison. The result-saving function (**Figure 15E**) allows the recognition results with bounding boxes to be saved directly to the mobile photo album for subsequent recording, presentation, and comparison.

The application package size was 34.5 MB, and the runtime dynamic memory usage was less than 80 MB. To evaluate the mobile inference performance of the model, the application was deployed on iPhone 12 and iPhone 13 for on-device testing. Test images representing typical scenarios were selected to verify the recognition performance and operational efficiency of the application under different complex conditions. To reduce the random errors caused by single-run fluctuations, the inference latency for each image was measured by repeating the test 60 times and calculating an average value. It should be noted that the average inference time and average FPS were computed independently based on repeated measurements. Because FPS is the reciprocal of inference time in each individual test, the mean FPS is not necessarily equal to 1000 divided by the mean inference time.

Compared with the baseline YOLO26n, MPE-YOLO achieved a lower average inference time and higher average FPS on both the iPhone 12 and iPhone 13. Specifically, the average inference time of MPE-YOLO was reduced by 3.08 ms/image on iPhone 12 and by 3.88 ms/image on iPhone 13 (**Table 8**). These results indicated that the lightweight improvements reduced the inference cost of mobile devices and improved real-time on-device responsiveness, meeting the application requirements for rapid empty-cell detection in greenhouse maize plug trays.

**Table 8:** Performance evaluation of the Corn Empty-Cell Detector APP on different iPhone devices.

Device	OS version	Processor	RAM (GB)	ROM (GB)	Model	Average inference time (ms/image)	Average FPS
iPhone12	iOS 18.7.7	Apple A14 Bionic	4	128	YOLO26n	26.82	46.09
					MPE-YOLO	**23.74**	**54.62**
iPhone13	iOS26.4.2	Apple A15 Bionic	4	256	YOLO26n	25.81	45.13
					MPE-YOLO	**21.93**	**55.08**

Bold values indicate the best-performing results.

## 4 Discussion

This study differs from the existing missing-seedling detection tasks. Previous studies have mostly focused on missed sowing detection (Yan et al., 2023) or post-transplant missing-plant detection (Zhang et al., 2024), whereas this study focuses on empty-cell recognition in large-scale greenhouse maize plug trays, a task that aims to determine the status of each plug cell and therefore has specific visual and application requirements. Under greenhouse nursery conditions, neighboring leaves may extend into empty-cell regions, while substrate covering may obscure cell edges, thereby weakening the visual distinction between empty and non-empty cells. Therefore, the key difficulty of this task lies not only in detecting empty cells, but also in maintaining reliable recognition under complex greenhouse plug-tray backgrounds caused by neighboring-leaf occlusion and substrate-covered cell edges.

The experimental results show that MPE-YOLO improves detection performance while maintaining a lightweight model structure. This indicates that effective empty-cell recognition does not necessarily rely on increasing model depth or computational cost. Instead, the improvement is closely related to whether the model design matches the structural characteristics of maize plug-tray scenes, where plug trays have a regular spatial arrangement and empty-cell regions usually show a certain degree of morphological consistency even when partially occluded or covered by substrate. These characteristics make it possible to reduce redundant computation while preserving useful cell-level structural information. Therefore, the lightweight design in MPE-YOLO is not merely a general compression strategy, but a task-adapted design for visually complex plug-tray empty-cell recognition.

Compared with existing lightweight models in agricultural vision, MPE-YOLO differs mainly in the way lightweight design is coupled with the specific recognition requirements of maize plug trays. Previous studies have generally pursued lightweight model design from two perspectives: network-structure optimization and deployment-oriented acceleration. In YOLO-based agricultural detection models, lightweighting is commonly achieved by redesigning the backbone, neck, or detection head to reduce parameters and GFLOPs (Feng et al., 2025; Jin et al., 2025; Lu et al., 2025). In CNN-based lightweight approaches, model compression and acceleration strategies, including compact CNN design, filter pruning, quantization, TensorRT acceleration, and hardware-oriented CNN acceleration, have been used to improve inference efficiency (Li et al., 2021a, 2023; Sang et al., 2022). These strategies are effective for reducing computational cost and improving inference speed. However, most of them are designed for visible and relatively independent targets, such as seedlings, weeds, fruits, or isolated seeds. In contrast, maize plug-tray empty-cell recognition requires fine-grained localization within a dense and regular cell structure. Therefore, the contribution of MPE-YOLO lies not only in developing a lightweight model, but also in the task-oriented integration of lightweight feature extraction, efficient feature fusion, attention-guided recalibration, hard-sample learning, false-detection control in target-absent scenes, and mobile deployment for structured greenhouse plug-tray empty-cell recognition.

Nevertheless, this study still has several limitations. The current dataset was collected under specific maize varieties, tray types, seedling growth stages, substrate moisture states, and greenhouse conditions. Although evaluations under occlusion, substrate-covering, and empty-cell-free conditions were conducted, independent stratified analyses across different seedling growth stages, illumination conditions, and substrate moisture states were not performed. In addition, the current 4 × 4 cropping strategy may alter the sample distribution relative to real deployment conditions, including the proportions of empty cells, non-empty cells, edge cells, and target-absent trays. Although this patch-based strategy preserves local plug-cell structure, maintains sufficient resolution for empty-cell localization, and supports efficient mobile inference, complete tray-level automated inference and its integration with transplanting equipment or long-term nursery management workflows still require further validation under practical deployment conditions.

## 5 Conclusion

In this paper, we develop MPE-YOLO, a task-adapted lightweight detector based on YOLO26n for empty-cell recognition in greenhouse maize plug trays under complex backgrounds caused by neighboring-leaf occlusion and substrate-covered cell edges. By integrating DSConv for lightweight feature extraction, CGSB for efficient feature fusion, C3k2-EMA for attention-guided feature recalibration, and DF-Loss for dual-focusing loss optimization, the model is designed to address the boundary ambiguity and background interference that define this task. Across repeated tests, MPE-YOLO achieves average precision, recall, mAP@0.5, and mAP@0.5:0.95 values of 93.8% (+1.6 percentage points), 94.0% (+0.2 percentage points), 97.0% (+0.3 percentage points), and 86.6% (+0.1 percentage points), respectively, with only 1.51 M parameters and 3.6 GFLOPs, and an FPS of 574.3. Evaluations under occlusion, substrate-covering, and empty-cell-free conditions further confirm its robustness under complex greenhouse backgrounds and its ability to reduce false detections in target-absent scenes. The successful deployment of Corn Empty-Cell Detector on iOS devices demonstrates that MPE-YOLO can support portable, real-time, and non-destructive plug-tray quality monitoring.

Future work will collect larger and more diverse datasets to improve the generalization ability of MPE-YOLO under practical greenhouse conditions. An automated full-tray inference pipeline will also be developed to extend MPE-YOLO from patch-level empty-cell recognition to complete plug-tray quality assessment. Furthermore, the integration of MPE-YOLO with automated transplanting systems and nursery management workflows will be further explored to promote its practical application in large-scale greenhouse maize seedling production.

## Data Availability

The data analyzed in this study is subject to the following licenses/restrictions: The original datasets and images generated and analysed during this study are available from the corresponding author upon reasonable request. Requests to access these datasets should be directed to xiaoyingzhang@sxau.edu.cn.
